# Gait environments recognition via synaptic convolutional neural network with memory autapses

**DOI:** 10.1186/s12984-026-02071-x

**Published:** 2026-07-27

**Authors:** Jirou Feng, Shuai Hao, Junhwan Choi, Junhwi Cho, Wenyue Cui, Jung Kim

**Affiliations:** 1 Department of Mechanical Engineering, Korea Advanced Institute of Science and Technology, 34141 Daejeon, Republic of Korea; 2 Inner Mongolia Autonomous Region Key Laboratory for Ecological Environment Collaborative Intelligence, Inner Mongolia Normal University, Hohhot, 010022 Inner Mongolia China; 3 School of Control Science and Engineering, Dalian University of Technology, Dalian, 116024 Liaoning China

**Keywords:** Gait environments recognition, Neuromorphic computing, Synaptic, Deep learning

## Abstract

Exoskeleton systems face significant challenges in maintaining reliable gait prediction under dynamically changing walking environments. Conventional learning-based methods often suffer from limited adaptability and high computational latency, especially when deployed on low-power embedded hardware. A neuromorphic computing framework based on a synaptic convolutional neural network with memory autapses (SCN-MA) is introduced for real-time gait environment recognition. A structured gait experiment was conducted to collect pneumatic mechanomyography (pMMG) and stretch signals under multiple walking conditions, forming a multimodal dataset for training and evaluation. The framework adopts multiple binary gait classification models trained in parallel, which enhances modularity and scalability. A reinforcement learning based ensemble policy is then trained to integrate the outputs of these models for final decision-making. The complete system is implemented on the Zynq-based FPGA platform, enabling end-to-end prediction with low latency and high energy efficiency. Experimental results confirm improved accuracy and adaptability over baseline models, supporting the use of neuromorphic architectures in exoskeleton systems.

## Introduction

The development of exoskeleton systems has accelerated in recent years with the aim of enhancing mobility, rehabilitation, and physical augmentation [1]. These wearable robots rely on effective environment perception to ensure safe and adaptive control under various terrain conditions. Transitions between level ground, ramps, and stairs are particularly critical and risky phases in gait rehabilitation, where falls and near-falls frequently occur. These risks are especially pronounced for stroke survivors, individuals with incomplete spinal cord injury (SCI), and lower-limb amputees using powered exoskeletons or orthoses. Gait environment prediction therefore plays a critical role in enabling timely adjustment of actuation strategies, which directly influences user comfort, energy efficiency, and injury prevention [2, 3]. However, acquiring high-quality biosignals that reflect environmental transitions remains challenging [4]. In addition, the differences between walking conditions often lead to ambiguous signal distributions, which complicate the classification. Achieving real-time prediction in wearable systems further requires computational frameworks that support efficient neural computation within the constraints of embedded hardware platforms. These challenges highlight the necessity of scalable and low-latency approaches that can be deployed for reliable environment-aware control in practical exoskeleton applications [5]. Therefore, the ability to anticipate upcoming walking environments and switch assistance modes in advance is clinically meaningful, providing safer and more adaptive locomotion support during rehabilitation and daily ambulation.

Traditional biomechanical data acquisition systems are often constrained by hardware rigidity, limited computational efficiency, and dependence on controlled laboratory settings [6]. These limitations reduce the ecological validity and practicality of many gait analysis studies. Recent advancements in wearable motion capture technologies, especially the emergence of sensor-integrated suits designed for daily and field use, have highlighted the growing importance of mobile and context-aware systems in real-world biomechanical data collection [7]. These developments offer new possibilities for capturing human movement with higher continuity, adaptability, and wearability [8, 9]. Conventional gait recognition methods typically rely on handcrafted features extracted from biomechanical or inertial sensor data [10]. While deep learning approaches such as convolutional neural networks [11], recurrent models like long short-term memory [12], and attention-based Transformer architectures [13] have shown promising accuracy under structured conditions, their performance often degrades when exposed to unstructured or previously unseen environments. Moreover, these architectures demand substantial computational resources, which limits their deployment on low-power embedded platforms typical in wearable robotics [14]. This mismatch between algorithmic complexity and hardware constraints becomes particularly problematic in scenarios where rapid environmental adaptation is essential. Consequently, these observations naturally prompt the following questions: (1) How can a gait classification dataset be constructed that is both easy to measure and exhibits high inter-condition separability? (2) What kind of neural computing model can achieve efficient real-time prediction of gait environments? (3) How can the proposed model be deployed on hardware to enable fully integrated end-to-end inference? These questions are driven by the increasing demands for adaptive locomotion support in wearable systems and the lack of scalable methods that bridge signal acquisition, learning, and real-time deployment. Therefore, developing a neuromorphic framework for environment-aware gait recognition presents both an inevitable and intriguing area of study.

Motivated by these challenges, a neuromorphic computing framework is proposed for achieves accurate prediction performance on the collected multimodal dataset, which integrates a biologically inspired synaptic convolutional neural network with memory autapses (SCN-MA) and an adaptive ensemble strategy for robust gait environment recognition. The contributions of this work are summarized as follows:

1. Constructed a gait environments recognition dataset: This dataset consists of pneumatic mechanomyography (pMMG) and stretch sensor signals collected from ten participants under five distinct walking conditions. The signal acquisition setup ensures noncontact sensing and consistent data quality across users and trials, facilitating robust environment recognition.
2. Proposed a synaptic convolutional neural network with memory autapses: The model adopts a parallel architecture where multiple binary classifiers are trained independently, each responsible for distinguishing between a specific pair of gait conditions. This modular design reduces task complexity for each submodel and improves scalability for future additions to the environment.
3. Introduced an ensemble strategy to aggregate the classifiers’ outputs: A reinforcement learning based policy is trained to dynamically select and combine predictions, improving the robustness of systems under varying signal distributions.
4. Deployed the proposed framework on a Zynq-based field-programmable gate array (FPGA) platform: The full system supports end-to-end gait environment prediction with low latency and power consumption, enabling real-time operation on embedded devices suitable for wearable exoskeletons.

The remainder of this paper is organized as follows. Section Related works reviews the relevant literature on gait recognition and neuromorphic computing. Section Methodology details the proposed methodology. Section Experiments and results presents the experimental setup and results. Section Discussion discusses the findings, implications, and limitations of this study, which are followed by the conclusions in Section Conclusions.

## Related works

### Gait environments recognition overview

Accurate gait environment prediction is fundamental to adaptive exoskeleton control, requiring both effective data acquisition and robust classification methods. Recent studies have explored various sensing modalities and algorithms to improve prediction performance under real-world conditions. Wang et al. [15] proposed a method based on shank-mounted inertial measurement units (IMU) to extract spatial-temporal and kinematic parameters, though the method is limited by weak environmental specificity. Dong et al. [16] developed a device-free approach using footstep-induced floor vibrations combined with contrastive learning, which avoids wearable sensors but suffers from environmental noise sensitivity. Zhang et al. [17] applied multi-MEMS inertial sensors with time-domain feature analysis and support vector machine (SVM) to achieve high-accuracy phase classification, yet the system depends on multiple sensor placements and controlled settings. Computer vision-based gait pattern recognition has also been explored for flat-fall prediction [18], providing a non-contact sensing alternative but facing potential limitations related to camera viewpoint, lighting conditions, and occlusion.

In terms of prediction models, Dong et al. [19] introduced a low-cost framework that integrates multi-source sensor fusion with lightweight classifiers such as SVM and convolutional neural network (CNN), achieving high accuracy with reduced computational cost but showing limited generalization across locomotion modes. Zhang et al. [20] proposed a method combining a long short-term memory (LSTM) network and dual adaptive frequency oscillators for gait phase estimation in continuous multi-locomotion tasks, which improved adaptability but relied on complex modules and multiple sensors. He et al. [21] employed a dual-branch Swin Transformer with asymmetric attention for radar-based gait recognition, achieving high precision but presenting challenges for embedded implementation due to hardware demands. Zhang et al. [22] addressed fall-related gait pattern recognition using surface electromyography (sEMG) and a hybrid neural network with transfer learning, which improved cross-subject adaptability but was not evaluated under diverse walking environments. Faisal et al. [23] developed a simple gait index using selected biomechanical parameters, offering interpretability but lacking adaptability to diverse environments.

Despite the progress in sensing technologies and prediction algorithms, several limitations persist. Current sensing systems often involve rigid placement, constrained laboratory settings, or intrusive configurations. Moreover, the generalization capability of learning-based models remains insufficient when faced with unseen walking environments or variations in terrain. Most deep architectures also demand high computational resources, limiting their real-time deployment on embedded platforms.

### Advances in neuromorphic computing

Neuromorphic computing has evolved from static artificial neural networks to biologically inspired models capable of capturing temporal dynamics. Spiking neural networks (SNNs) simulate brain-like information processing by encoding data through spike timing, offering natural advantages in modeling time-dependent signals [24]. Early reviews have shown that SNNs enable compact and efficient temporal representation, suitable for pattern recognition under dynamic conditions. Building on this, spike-based encoding methods such as first-spike latency coding have been applied in neuromorphic tactile systems to classify dynamic interactions with millisecond-level resolution [25]. These methods demonstrate fast and precise processing that aligns with the biological nervous system.

Further developments have introduced neuroscience-inspired synaptic plasticity mechanisms [26]. Experimental studies have revealed distinct phases of synaptic modification that are critical for memory formation, informing computational models that distribute learning across time and structure. Recent advances have incorporated self-backpropagating synaptic updates into SNNs and artificial networks, allowing local learning signals to propagate backward and thereby reducing computational load. To enable real-time operation, neuromorphic computing platforms have been developed for embedded applications [27]. These hardware systems process neural models at high speed and low energy consumption, making them ideal for robotics and wearable intelligence. Brain-inspired architectures that combine conventional and neuromorphic components have been successfully applied to place recognition [28] and human activity classification [29], achieving low latency and high accuracy on specialized chips.

These works demonstrate the potential of neuromorphic models and hardware for adaptive perception. However, an integrated framework that combines efficient spike-based modeling, biologically grounded learning, and embedded deployment for locomotion recognition tasks remains lacking.

## Methodology

**Fig. 1:**
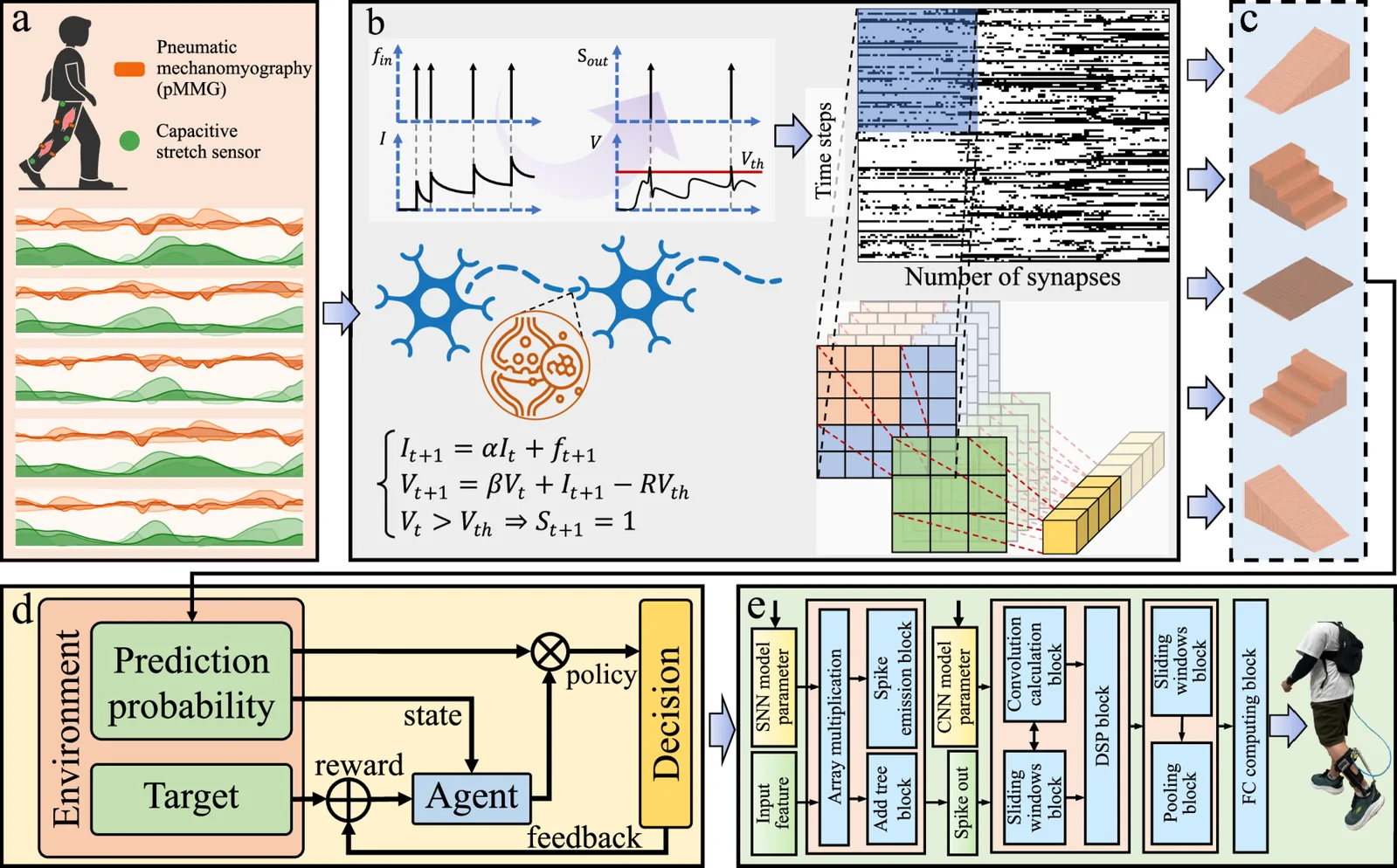
Overview of the proposed neuromorphic computing framework. **a** Construction of the gait environment dataset: Pneumatic mechanomyography (pMMG) and capacitive stretch sensor signals are collected from sensors attached to the lower limb. The multimodal biosignals are synchronously recorded while participants walk across different environmental conditions. **b** Architecture of the proposed SCN-MA: Spike-based temporal inputs are processed through synaptic dynamics, where current and membrane potential evolve according to biologically inspired differential equations. Spatiotemporal patterns are encoded across multiple synaptic channels and processed using convolutional filters, enabling dynamic representation of input signals. **c** Gait environment classification output: Each binary classifier is trained to distinguish between a specific pair of walking conditions, including level walking, stair ascent/descent, and ramp ascent/descent. This modular structure allows scalable and targeted classification. **d** Reinforcement learning–based ensemble strategy: A decision agent receives classifier predictions and dynamically selects the final decision based on state and reward feedback from the environment, thereby optimizing the policy for robust classification. **e** Hardware implementation: The complete system is implemented on a field-programmable gate array (FPGA), integrating spike encoding, convolution computation, and fully connected layers. Real-time prediction is supported for potential integration with wearable exoskeleton systems

To address the above limitations, a neuromorphic computing framework is proposed for gait environment recognition based on the synaptic convolutional neural network with memory autapses and hardware-aware model deployment, which is illustrated in Fig. 1.

### Collection of gait environment dataset

To evaluate the proposed gait environment recognition framework under realistic conditions, a dedicated dataset was collected comprising five representative walking environments. In our setup, non-contact sensing modalities were utilized, where a pMMG sensor captured variations in muscle activity and a capacitive stretch sensor monitored changes in joint angles. By integrating these complementary signals, a multimodal dataset was constructed to capture both muscular dynamics and joint kinematics, enabling a robust characterization of locomotion in diverse gait environments.

#### pMMG and soft stretch sensor fabrication

**Fig. 2:**
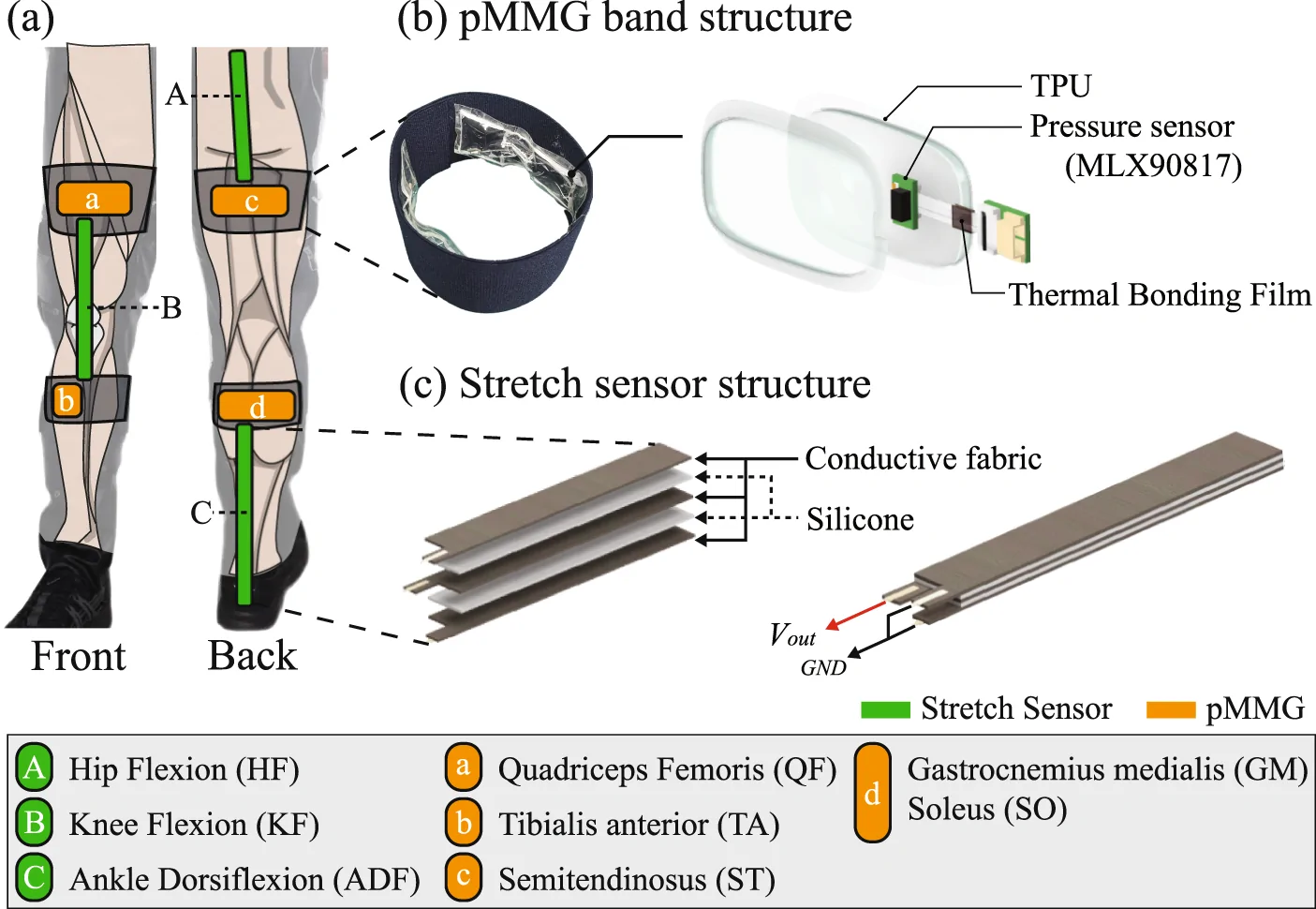
Sensor setup and structural design. **a** Placement of the pMMG and stretch sensors on the lower limb. **b** Structure of the pMMG sensor with integrated absolute pressure sensor. **c** Structure of the capacitive stretch sensor illustrating its layered configuration

The pMMG band was fabricated to detect muscle activity by measuring subtle pressure changes induced by muscle swelling during contraction. Unlike traditional surface electromyography (sEMG), which relies on electrical potentials on the skin surface and is highly sensitive to noise and electrode placement, pMMG directly measures the mechanical activity of muscles without requiring direct skin contact. In our previous research, pMMG was shown to exhibit a highly linear relationship between muscle force and measured pressure, while also demonstrating high repeatability and accuracy in hand gesture recognition tasks [30–32]. The pMMG sensor integrates an MLX90817 absolute pressure sensor (Melexis, Belgium) into the custom-designed air sac, and the sensor structure is illustrated in Fig. 2b. The sac is fabricated from thermoplastic polyurethane (TPU) film, a material selected for its softness, durability, and high tensile strength, thereby ensuring user comfort and long-term reliability. The TPU layers are sealed using high-frequency welding, which produces a strong, airtight bond. Furthermore, a thermoplastic adhesive film is employed to attach the TPU film securely to the flexible flat cable (FFC), providing both mechanical stability and a reliable electrical connection.

With high linearity, good repeatability, and low hysteresis, the capacitive stretch sensor enables reliable long-term monitoring of joint kinematics and locomotion [33, 34]. Its working principle is based on capacitance changes between two conductive fabric electrodes separated by a dielectric layer, where stretching increases the sensor length and decreases the dielectric thickness, resulting in a measurable capacitance variation. Based on our previous work [35], we fabricated a fabric-based soft stretch sensor with a five-layer design, as illustrated in Fig. 2c. The sensor adopts a sandwich structure composed of three layers of conductive fabric and two layers of dielectrics, with an overall dimension of 10 mm *× * 90 mm *× * 3 mm. The conductive fabrics (TB3333F, Threebond, Japan) were bonded using a 500 *μ *m silicone rubber layer (Ecoflex 00-30, Smooth-On, USA). The middle conductive layer was connected to the signal line, while the outer layers were assigned as ground. To reduce interference from external signals and enhance durability, the outermost layers were encapsulated with silicone rubber.

#### Sensor setup

To capture muscle activity during walking, pMMG sensors were positioned over the major lower-limb muscles, namely the Quadriceps Femoris (QF), Semitendinosus (ST), Tibialis Anterior (TA), Gastrocnemius Medialis (GM), and Soleus (SO) [36]. Considering the physiological characteristics of these muscles, sensors with a size of 200 mm *× * 100 mm *× * 10 mm were fixed with elastic bands on the thigh to monitor QF and ST, and on the shank to monitor GM and SO, while a smaller sensor of 100 mm *× * 100 mm *× * 10 mm was used for TA. Stretch sensors were attached to the hip, knee, and ankle joints to capture walking kinematics. Their placement allowed the measurement of joint angle changes during locomotion, complementing the muscle activity signals acquired by the pMMG sensors. Their placement allowed the measurement of joint angle changes during locomotion, complementing the muscle activity signals acquired by the pMMG sensors. The overall sensor setup is illustrated in Fig. 2. The capacitance change of each soft stretch sensor was converted into a digital signal by calculating the discharge time using an NE555p timer (Texas Instruments, USA). For data collection, signals from all sensors were synchronously acquired using the myRIO-1900 (National Instruments, USA) at a sampling rate of 250 Hz.

#### Experimental protocol

In this study, five representative gait environments were designed to evaluate the proposed framework under diverse locomotion conditions, including level-ground walking, stair ascent, stair descent, ramp ascent, and ramp descent. These environments were selected to represent typical locomotion scenarios encountered in daily activities and rehabilitation training [37–39]. The angle of the ramp was set to $10^{\circ }$ [40], and the width and height of the stairs were 60 and 10 cm, respectively.

Ten healthy adults (nine males and one female) participated in the experiment. The participants had an average age of 26.1 ± 3.3 years, height of 172.8 ± 3.3 cm, and body weight of 68.8 ± 6.6 kg. All participants had no history of musculoskeletal or neurological disorders that could affect gait performance. Written informed consent was obtained from all participants prior to the experiment, and the study was approved by the Institutional Review Board of the Korea Advanced Institute of Science and Technology (KAIST) (KH2016-062, approved on May 3, 2023).

**Fig. 3:**
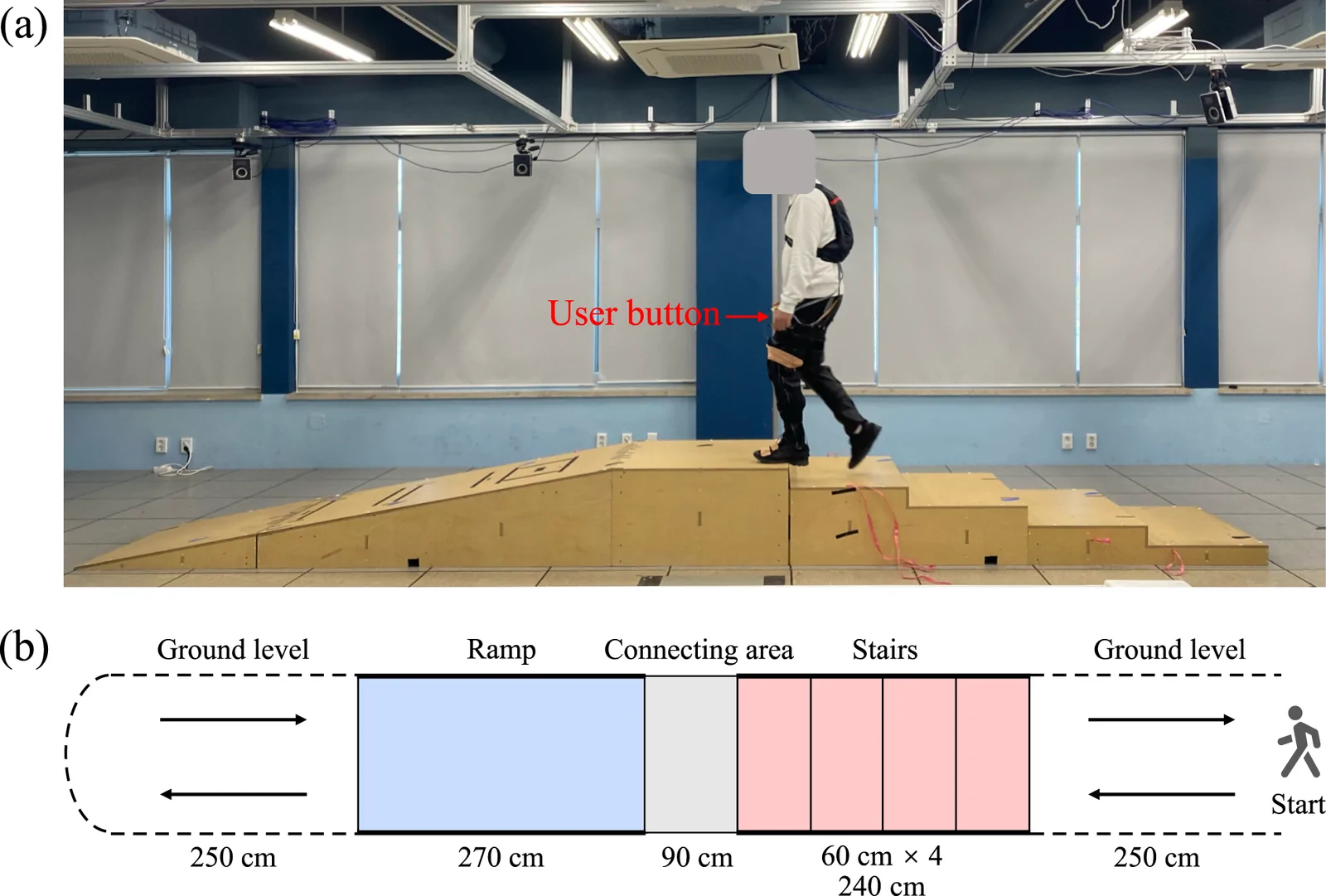
Experimental setup and walking course. **a** Photograph of the experimental setup and the user button used for environment labeling. **b** Schematic diagram of the walking course illustrating the sequence of gait environments

Before the experiment, each participant was instructed to stand upright to record the initial sensor values for calibration. After calibration, each participant completed 20 trials of a walking course consisting of multiple walking environments in a fixed order: beginning on level ground, followed by ramp ascent, stair descent, level ground, turning around and continuing on level ground, stair ascent, ramp descent, and finally ending on level ground. Each locomotion condition, except for level-ground walking, consisted of four steps corresponding to two gait strides. A user button was provided to all participants, who was instructed to press the button at the heel contact of the first step whenever transitioning to a new walking environment. The button signal was used for environment labeling during data processing. To prevent muscle fatigue, participants were given a 30-second rest between consecutive trials. The experimental setup and walking course are illustrated in Fig. 3.

### Synaptic convolutional neural network with memory autapse

SNNs provide a biologically plausible computing model capable of processing time-dependent signals with high temporal precision and low energy cost, making them well-suited for gait environment recognition tasks in wearable systems. To enhance temporal information representation, synaptic neuron dynamics are adopted in time series prediction rather than traditional leaky integrate-and-fire (LIF) models [41]. The synaptic model includes explicit modeling of synaptic current $I_t$ and membrane potential $V_t$, governed by the following update equations:

1 $$\begin{aligned} I_{t}= & \alpha I_{t-1}+f_{t} \end{aligned}$$

2 $$\begin{aligned} V_{t}= & \beta V_{t-1}+I_{t} \end{aligned}$$

3 $$\begin{aligned} S_{t}= & {\left\{ \begin{array}{ll} 1 & \text { if } V> V_{th} \\ 0 & \text { otherwise } \end{array}\right. } \end{aligned}$$

where *α * and *β * are decay constants, $f_{t}$ denotes the input current, $V_{th}$ is the firing threshold, and $S_{t}$ is the binary spike output. Compared to LIF models, synaptic dynamics enable more nuanced integration of temporal input and support a richer encoding of spike-based activity.

**Fig. 4:**
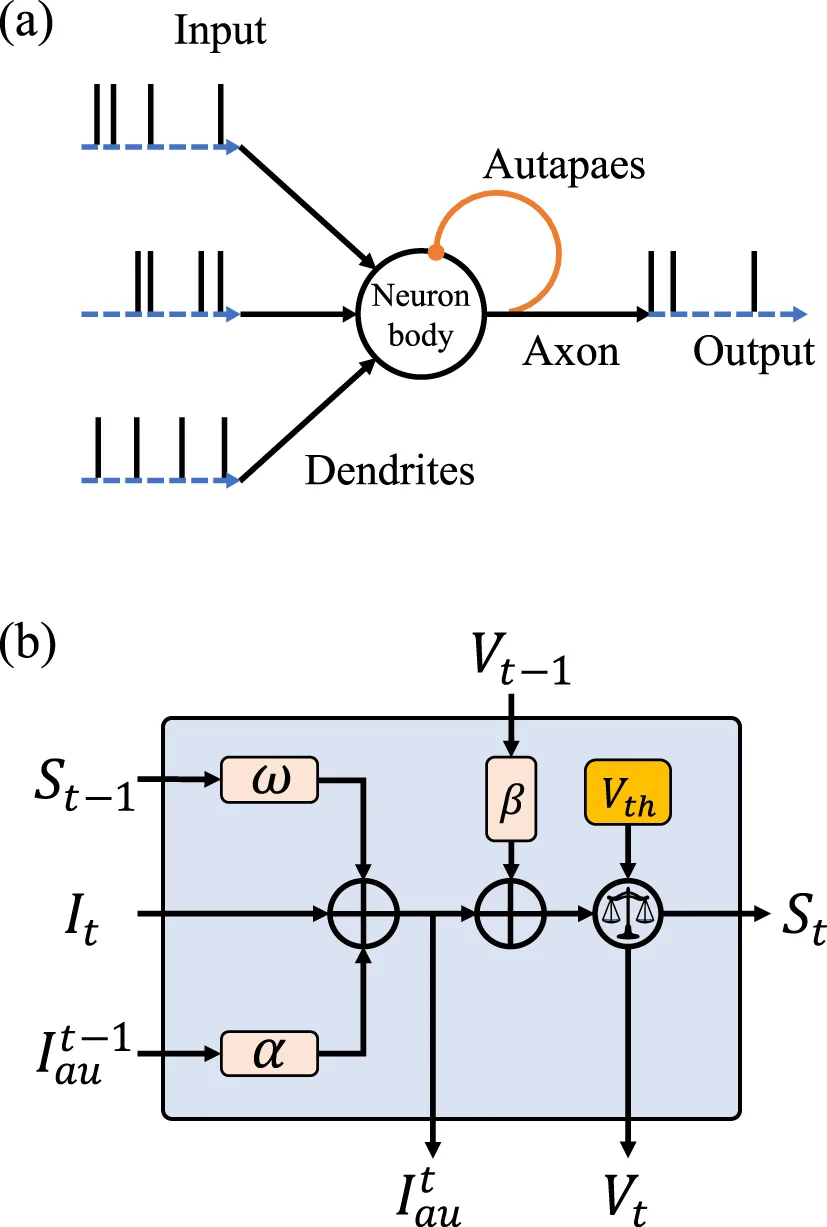
Illustration of synaptic dynamics with autapse. **a** Biological basis of autapse: The neuron receives spike inputs on its dendrites and feeds back its own output via an autaptic connection to introduce memory. **b** Calculation process of synaptic with autapse. The neuron updates synaptic current and membrane potential using prior spike output and autaptic current $I_{au}$, incorporating dynamic memory into the temporal integration process

**Fig. 5:**
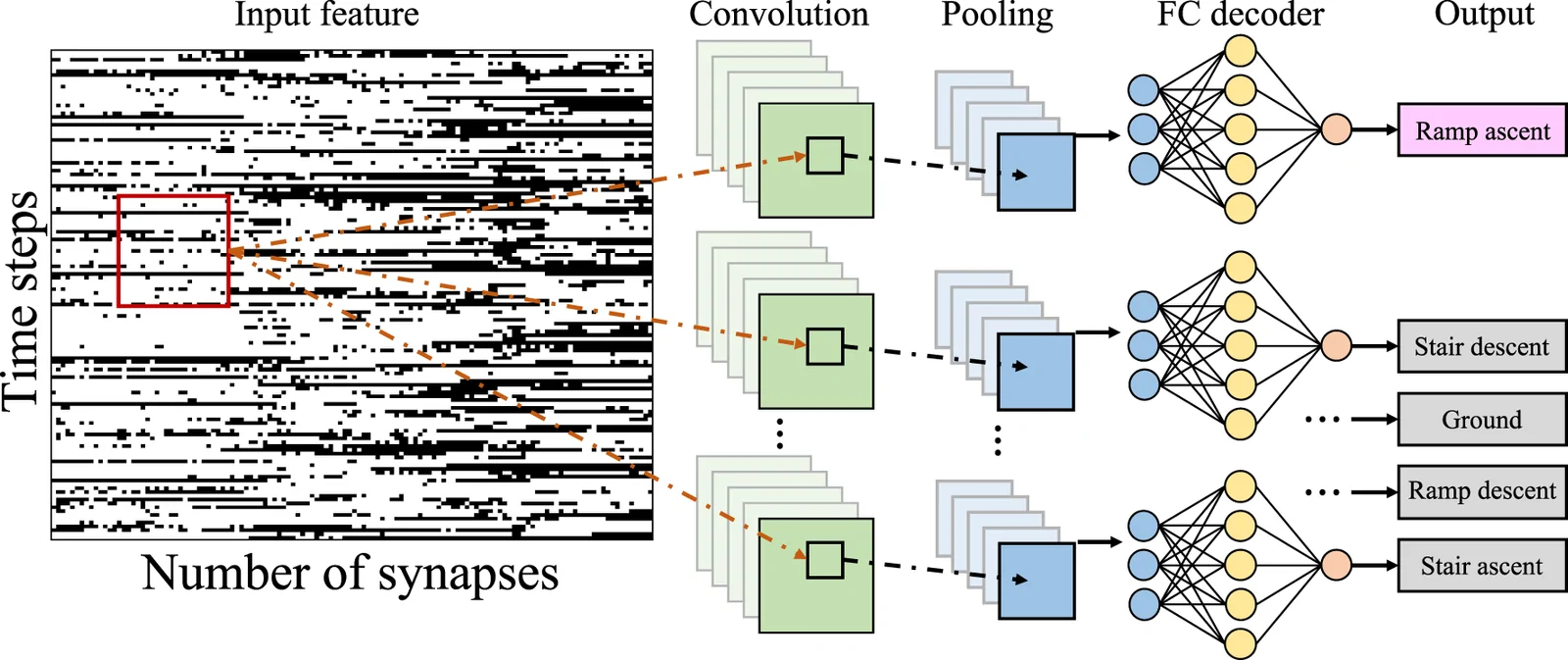
Spike-based convolutional classification framework. The input feature is a spatiotemporal binary matrix representing spike activity across synapses and time. Convolutional filters extract local temporal patterns, followed by pooling and classification through fully connected layers. Each classifier distinguishes between two gait conditions, and their outputs are integrated by a reinforcement learning agent to generate the final prediction

Inspired by findings in cortical neuroscience, the model incorporates an autapse mechanism, in which a neuron forms synaptic contact with its own dendritic arbor. As reported by Yin et al. [42], autapses have been identified in pyramidal cells and shown to amplify burst firing and enhance coincidence detection. To simulate this effect, an additional synaptic current term is introduced based on prior outputs, which is shown in Fig. 4. The synaptic dynamics with autapse model expands the dynamics as follows:

4 $$\begin{aligned} I^{t}_{au}= & \alpha I^{t-1}_{au}+\omega S_{t-1}+f_{t} \end{aligned}$$

5 $$\begin{aligned} V_{t}= & \beta V_{t-1}+I^{t}_{au} \end{aligned}$$

6 $$\begin{aligned} S_{t}= & {\left\{ \begin{array}{ll} 1 & \text { if } V> V_{th} \\ 0 & \text { otherwise } \end{array}\right. } \end{aligned}$$

where $I^{t}_{au}$ represents the autaptic current, *α * controls its decay over time, *β * represents the membrane potential decay rate, and *ω * corresponds to the autaptic recurrent weight that scales the contribution of the previous output spike to the current neuronal state. This mechanism enables the neuron to maintain a form of short-term memory, allowing prior spike outputs to influence subsequent integration and decision-making. In the experiments, sensor signals are encoded into spike trains with 128 input neurons firing over 100 discrete time steps. Following spike encoding and integration, the output spike matrix is passed into a lightweight convolutional decoder, which is shown in Fig. 5. This decoder uses temporal-spatial convolution to extract features, followed by pooling and a fully connected layer for binary classification. The decoding process is defined as:

7 $$\begin{aligned} y=\sigma \left( W_{fc} \cdot pool\left( conv\left( S \right) \right) +b \right) \end{aligned}$$

where $conv\left( \cdot \right)$ denotes convolution, $pool\left( \cdot \right)$ represents pooling, $W_{fc}$ is the weight matrix of the fully connected layer, *b* is the bias term, and $\sigma \left( \cdot \right)$ is the sigmoid activation function producing binary class probabilities.

To aggregate multiple binary classifiers into a unified prediction, an ensemble policy is learned using reinforcement learning. At each time step, the agent receives the state vector composed of the output probabilities $p_i$ from each classifier and selects an action $a_{t} \in \left\{ 1, 2, \dots , K \right\}$ representing the final gait class:

8 $$\begin{aligned} a_{t} = arg \max _{\pi } \mathbb {E} \left[ r_{t} \mid P_{t},\pi \right] \end{aligned}$$

where *π * is the learned policy, $P_{t}=\left[ p_1, \dots ,p_N \right]$ is the state, and $r_{t}$ is the classification reward. The agent learns to maximize expected rewards by aligning its predictions with the ground truth class in each environment.

### System architecture

**Fig. 6:**
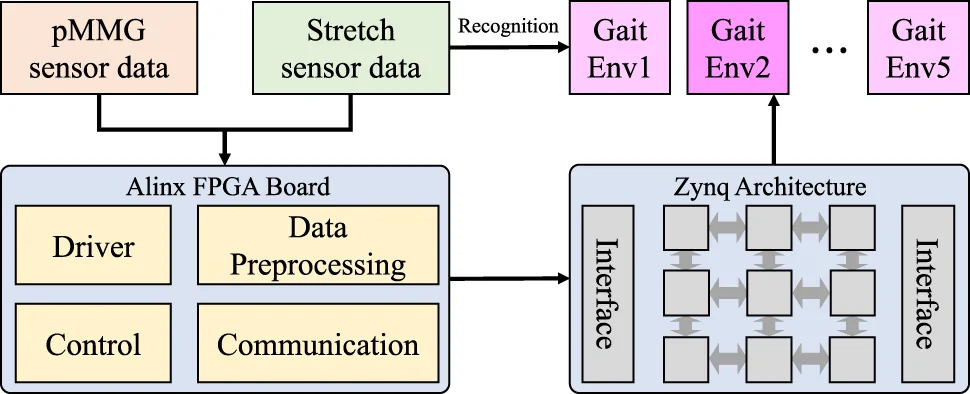
System workflow from signal acquisition to hardware-level gait prediction. The pipeline begins with pMMG and stretch sensors capturing multimodal gait information. Data are then preprocessed on the Alinx FPGA board through driver, control, communication, and processing modules. Gait predictions are generated using a neuromorphic model mapped onto a parallel computing array within the Zynq architecture

To enable real-time gait prediction and efficient neuromorphic inference, the proposed SCN-MA model is deployed on a heterogeneous hardware platform based on Zynq architecture, using the AX7Z100B development board (Alinx, China). The system integrates a processing system (PS) for control logic and a programmable logic (PL) section for computational acceleration. Figure 6 illustrates the overall system workflow from multimodal signal acquisition to gait prediction and hardware implementation.

The PL section implements a dedicated neuromorphic accelerator. It includes a sparse convolutional processing module optimized for event-driven spikes, a memory-efficient synaptic current integration unit, and a lightweight CNN-based decoder. Synaptic dynamics and membrane potential updates are modeled using pipelined logic to simulate biological integration and firing. A sparse computing pipeline is introduced to process only non-zero spikes, thereby improving computational efficiency and reducing energy consumption. The synaptic convolution modules utilize kernel logic and coordinate-based memory mapping to enable simultaneous updates of multiple neurons. A memory reuse strategy is adopted for high-throughput data streaming across layers. For data encoding and decoding, a dual-port Block RAM (BRAM) structure is used. Spiking data are encoded using coordinate-and-mask compression formats to reduce memory bandwidth. Convolution and pooling operations are performed using a weight reuse scheme with optimized accumulation modules. Post-convolution activations are processed through CNN layers implemented with Digital Signal Processing (DSP) slices and quantized integer arithmetic, ensuring low-latency inference. The final output of each binary gait classifier is stored in a probability register array and forwarded to a reinforcement learning–trained ensemble policy. This policy operates on the PL section using a lightweight lookup-based mechanism that combines classifier outputs based on learned weights.

The architectural mapping ensures that each model component is seamlessly integrated into the hardware execution pipeline. These design strategies yield a highly efficient embedded system capable of performing neuromorphic gait prediction in real time. In summary, the proposed hardware design bridges the gap between computational neuroscience and embedded control, enabling the deployment of biologically inspired models in wearable robotic systems. The effectiveness and efficiency of this implementation are quantitatively validated in the following section.

## Experiments and results

To evaluate the proposed framework, experiments were conducted using the constructed multimodal gait environment dataset. This section first presents the structure of the dataset. The performance of the SCN-MA model is then examined in this dataset, including its predictive capacity and deployment behavior on the hardware platform.

### Dataset construction

**Fig. 7:**
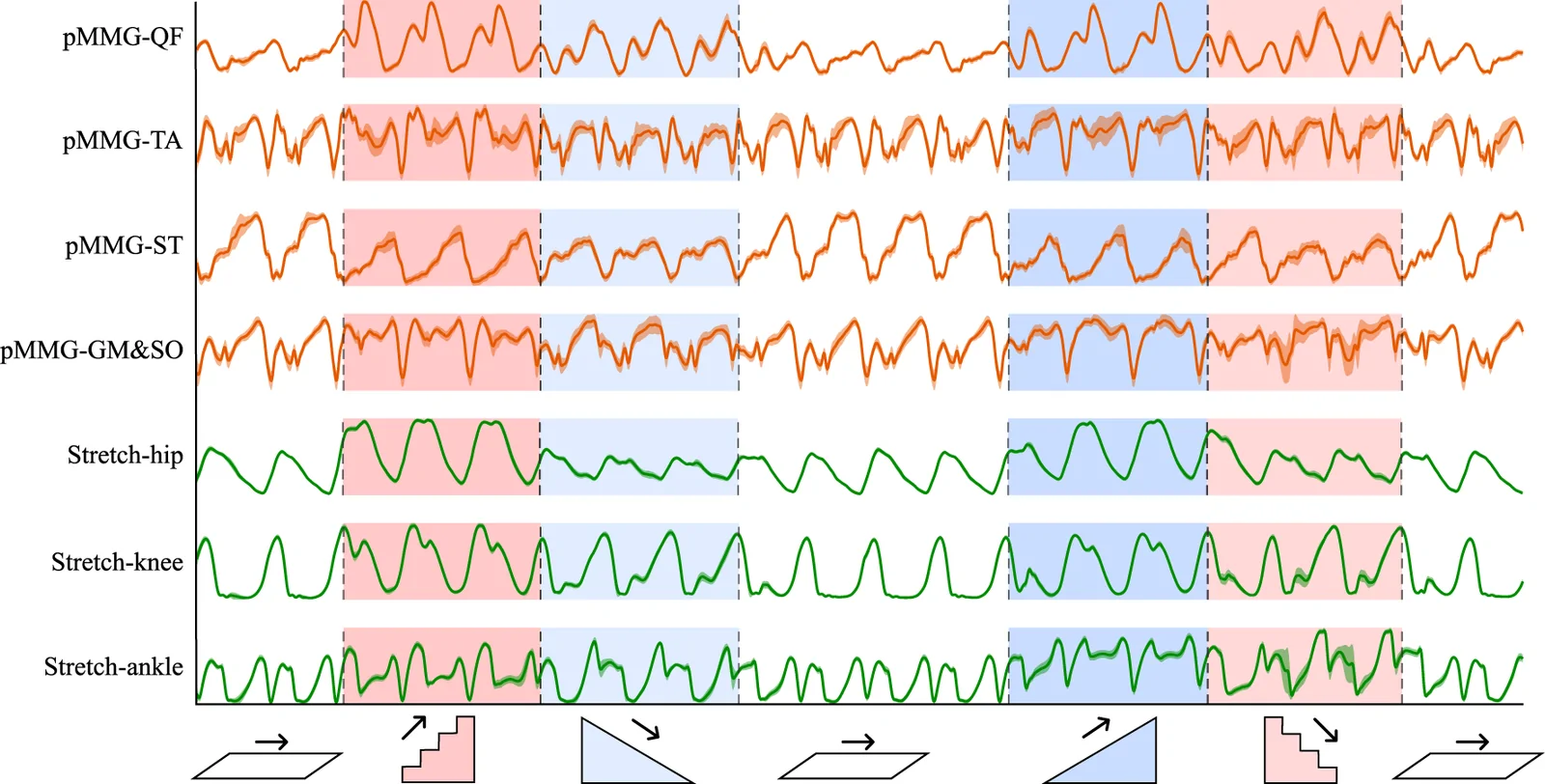
Averaged sensor signals from Subject 1 over 20 walking trials after normalization to the gait cycle. The shaded regions represent the standard deviation across trials

The dataset includes synchronized measurements from four pMMG sensors and three capacitive stretch sensors, capturing both muscle activity and joint kinematics across five gait environments: level-ground walking, ramp ascent, stair descent, ramp descent, and stair ascent. Since the pMMG sensors capture muscle volume changes during motion, the pMMG signals were calibrated using their initial values, and the recorded data represent the relative value changes over time. All signals were sampled at 250 Hz, with each participant performing 20 walking trials as described in the experimental protocol. Each trial contained continuous recordings for all gait environments in sequence, resulting in continuous multimodal recordings covering all five environments. Figure 7 illustrates the averaged signals from Subject 1 over 20 walking trials after normalization to the gait cycle. The shaded regions indicate the standard deviation across the 20 trials.

No manual feature extraction was applied; the raw signals were directly used for spike encoding in subsequent processing. For model training and evaluation, the dataset was divided into training and testing sets with an 8:2 ratio, where four trials were randomly selected from each participant as test data, and the remaining 16 trials were used for training.

### Model evaluation

The multimodal gait dataset described in the previous section is used for model evaluation. Each binary classifier in the proposed SCN-MA framework is trained to distinguish between two specific walking conditions. The input signals are first encoded into spike trains and then processed by the synaptic convolutional model with memory autapse. The key hyperparameters of the proposed model are summarized in Table 1.

For performance comparison, four representative baseline models, namely CNN, LSTM, SNN, and Transformer, are included in the evaluation. To evaluate the classification performance of each binary gait model, three standard metrics are adopted: precision (*Pre*), recall (*Rec*), and F1 score ($F_1$). These metrics are defined as follows:

9 $$\begin{aligned} Pre= & \frac{TP}{TP+FP} \end{aligned}$$

10 $$\begin{aligned} Rec= & \frac{TP}{TP+FN} \end{aligned}$$

11 $$\begin{aligned} F_1= & \frac{2\times TP}{2\times TP+FP+FN } \end{aligned}$$

where *TP*, *FP*, and *FN* denote true positives, false positives, and false negatives, respectively. These indicators provide a comprehensive evaluation of model correctness and class-specific performance.

**Table 1:** Key hyperparameters of the proposed SCN-MA

Hyperparameter	Value
Synaptic current decay constant	0.9
Membrane potential decay constant	0.85
Spike threshold voltage	1
Autapse current decay constant	0.8
Number of input spike encoders	128
Convolution kernel size	3*× *3
Learning rate	0.0001
Batch size	64
Optimizer	Adam

**Fig. 8:**
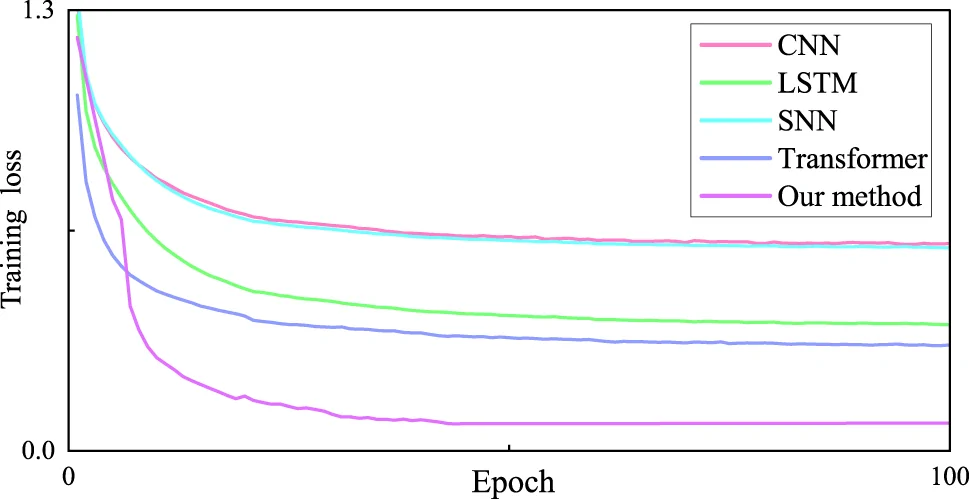
Training loss comparison of different models on Subject 1. The proposed SCN-MA exhibits the fastest decrease in training loss with the smoothest convergence trend among all compared methods, indicating superior training stability and optimization efficiency

In addition to the classification metrics, the training behaviors of different models are further compared using training loss curves, so that convergence speed and optimization stability can also be examined during learning. As shown in Fig. 8, the proposed SCN-MA exhibits faster loss reduction and a smoother convergence trajectory than the baseline models, indicating better training stability and optimization efficiency.

**Fig. 9:**
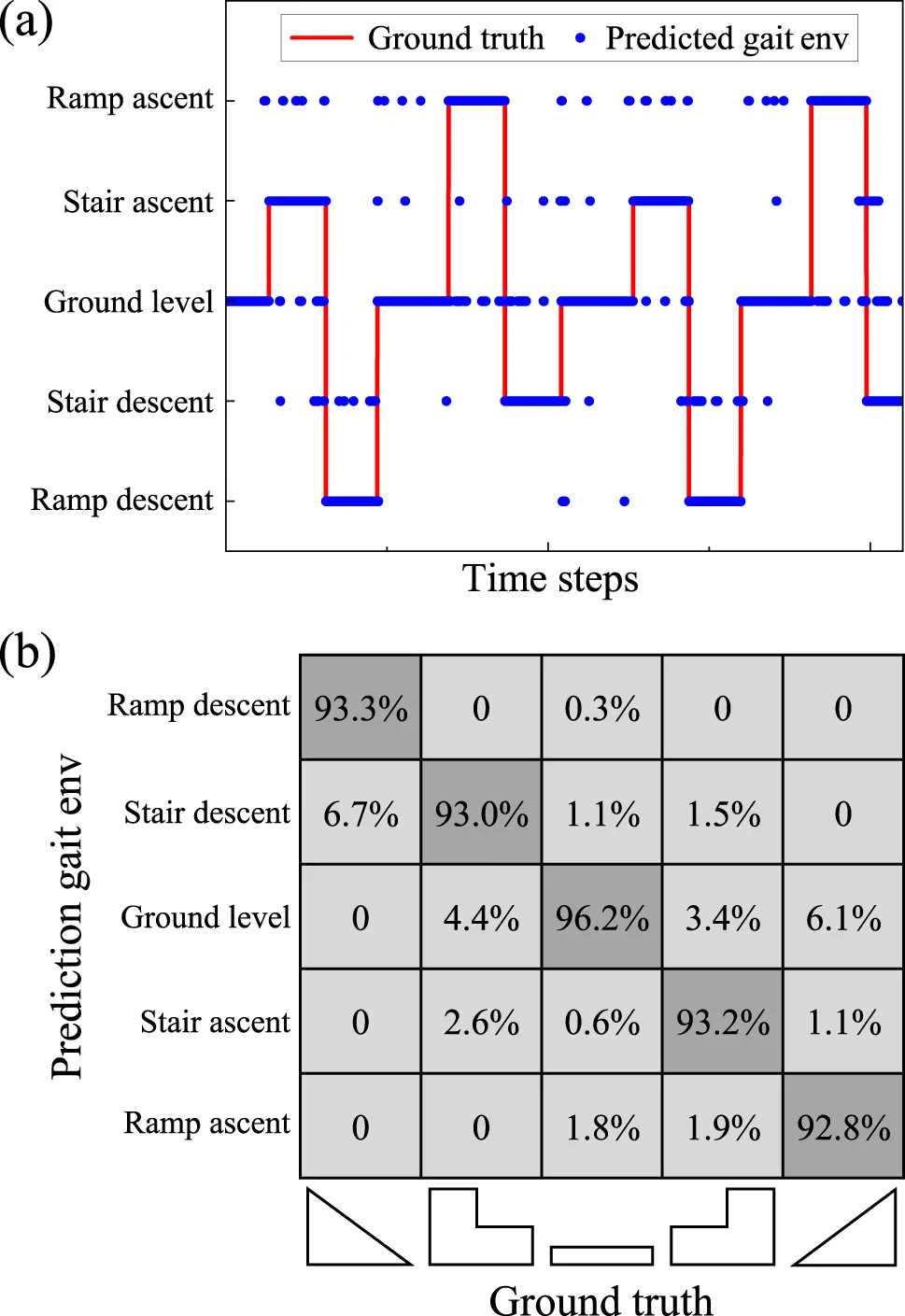
Gait classification performance on Subject 1. **a** Predicted and ground truth gait labels over time. The alignment demonstrates the effectiveness of the SCN-MA model in capturing environment transitions with temporal precision. **b** Confusion matrix across five terrain conditions. The diagonal dominance reflects high classification accuracy, with minor confusion between adjacent ramp and level categories

The classification performance of the SCN-MA model was first evaluated on Subject 1. As shown in Fig. 9a, the predicted gait environment labels closely follow the ground truth over time. The strong alignment between predicted and actual transitions demonstrates the model’s ability to capture gait phase shifts and terrain changes with high temporal resolution. This result highlights the effectiveness of spike-based encoding and synaptic integration in preserving the dynamic characteristics of biosignals. The detailed prediction distribution is presented in Fig. 9b as a confusion matrix, where each row represents the model’s prediction, and each column corresponds to the true gait category. Most samples lie along the diagonal, indicating clear separability of their signal patterns across the five gait environments. The most pronounced asymmetric confusion occurs from ramp descent to stair descent (6.7%), likely due to shared downhill dynamics. Minor misclassifications are observed between ramp ascent and level ground, which may be attributed to overlapping signal morphology during inclined walking with minimal elevation change. Overall, the matrix reflects high precision with class-specific nuances that align with biomechanical similarities.

**Fig. 10:**
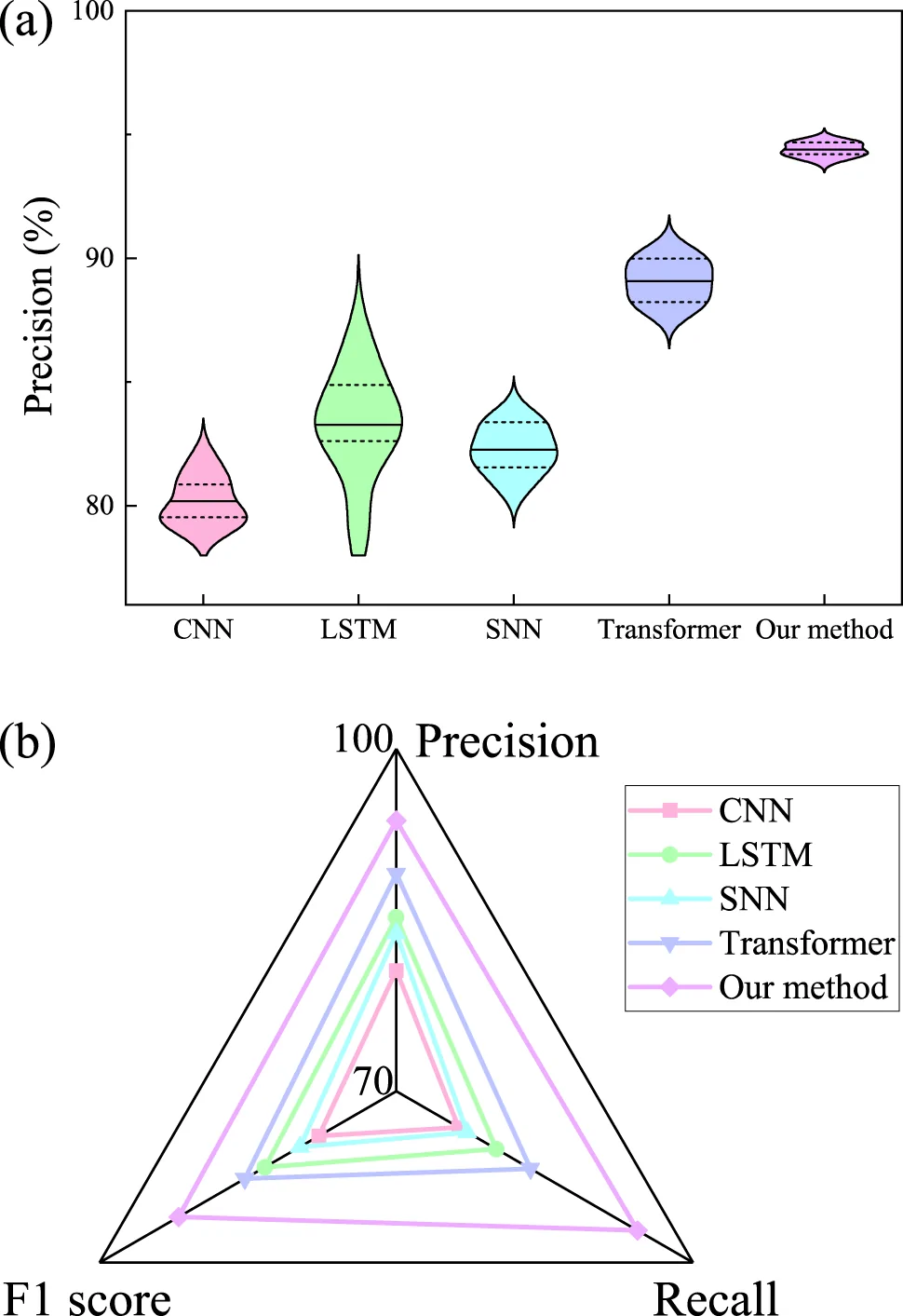
Metric comparison of different models on Subject 1. **a** Violin plot of prediction precision across CNN, LSTM, SNN, Transformer, and SCN-MA. The proposed model achieves the highest average precision with the lowest variance. **b** Radar chart illustrating precision, recall and F1 score across all models. SCN-MA forms the largest enclosed area, indicating superior and balanced classification performance

To further analyze the model’s classification behavior, Fig. 10a presents violin plots comparing the precision scores of CNN, LSTM, SNN, Transformer and the proposed SCN-MA. Among them, SCN-MA shows not only the highest average precision but also the narrowest distribution, indicating more stable prediction quality. This advantage stems from the model’s use of synaptic dynamics and autapse-enhanced memory, which together enhance sensitivity to temporal correlations and reduce spurious activations. Figure 10b provides a radar chart comparison over three metrics: precision, recall, and F1-score. The proposed model consistently scores higher across all dimensions, enclosing the largest area on the plot. This suggests that SCN-MA achieves a more balanced tradeoff between positive prediction accuracy and coverage, which is particularly important in real-time multi-condition gait recognition.

**Fig. 11:**
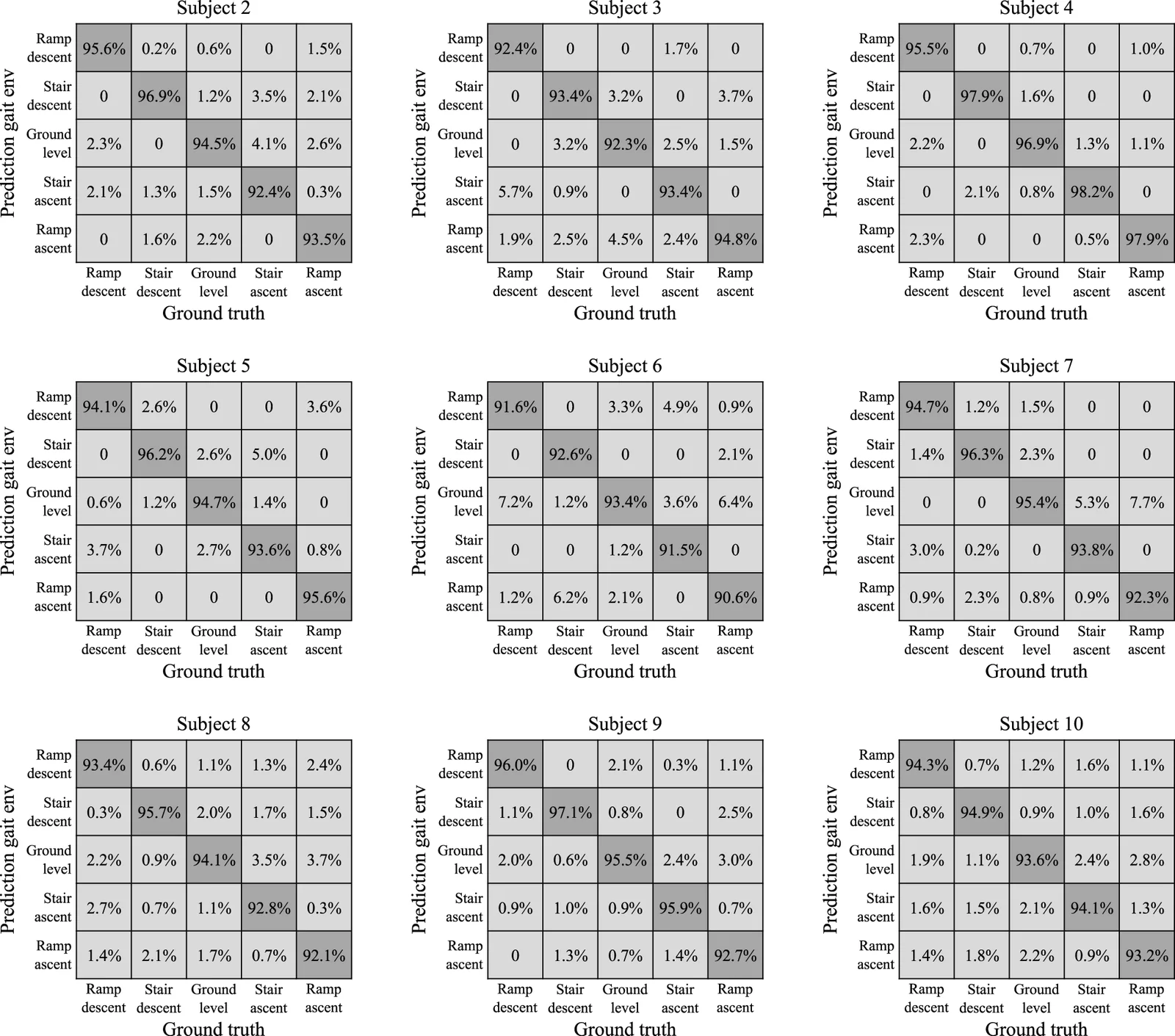
Confusion matrices across other subjects. Each subfigure presents the predicted versus actual gait environment labels for an individual subject. The consistently dominant diagonals across all confusion matrices reflect the model’s robustness to inter-subject variability and stable generalization across different walking patterns

To validate generalization, additional experiments were conducted on data from Subject 2 to Subject 10. Figure 11 shows the confusion matrices for these subjects. The performance remains consistent across individuals, with stair-related gaits showing the most distinct predictions. Although a few subjects exhibit minor confusion between adjacent ramp and ground conditions, the primary diagonal remains dominant. This demonstrates the model’s robustness to subject-specific variability and sensor placement differences.

**Table 2:** Results of comparative experiment for different subjects

Metrics	Precision (%)					Recall (%)					F1 score (%)				
Method	CNN	LSTM	SNN	Transformer	Ours	CNN	LSTM	SNN	Transformer	Ours	CNN	LSTM	SNN	Transformer	Ours
Subject 2	80.86	86.44	83.39	90.17	94.57	76.32	80.12	77.18	83.57	92.84	77.83	83.28	79.72	85.32	93.67
Subject 3	79.53	83.62	81.55	87.90	93.28	71.36	79.92	75.36	81.07	90.82	75.28	82.92	77.11	83.32	91.84
Subject 4	83.57	79.29	82.23	89.99	97.29	78.51	82.98	79.67	85.49	96.20	80.03	85.42	78.37	86.96	96.73
Subject 5	79.14	82.94	80.80	88.23	94.87	72.24	78.13	76.38	83.24	92.77	73.05	81.08	75.14	84.13	93.72
Subject 6	80.82	85.02	81.89	89.12	91.94	73.24	80.13	77.35	80.57	91.25	76.05	84.08	76.89	82.26	91.51
Subject 7	81.84	83.44	85.07	88.67	94.51	76.81	79.01	74.52	84.23	93.84	78.58	81.17	74.43	82.58	94.15
Subject 8	80.08	84.59	81.51	90.46	93.83	73.31	77.00	77.06	79.50	95.38	74.09	86.09	76.78	83.94	92.08
Subject 9	78.95	79.83	81.64	87.21	95.76	71.61	79.03	78.03	79.82	91.67	73.22	80.00	77.25	81.67	91.19
Subject 10	80.74	83.99	84.22	88.32	94.03	72.52	78.57	77.38	82.55	94.24	76.59	85.11	78.94	82.86	92.79
Average	80.61	83.24	82.48	88.89	94.45	73.99	79.43	76.99	82.23	93.22	76.08	83.24	77.18	83.67	93.08

Table 2 summarizes the averaged classification performance for all models. The proposed SCN-MA model achieves the best performance across precision, recall, and F1 score on the ten-subject dataset. Compared with the best-performing baseline, SCN-MA improves average precision, recall, and F1 score by 5.56, 10.99, and 9.41 percentage points, respectively, corresponding to relative improvements of 6.3%, 13.4%, and 11.2%.

**Fig. 12:**
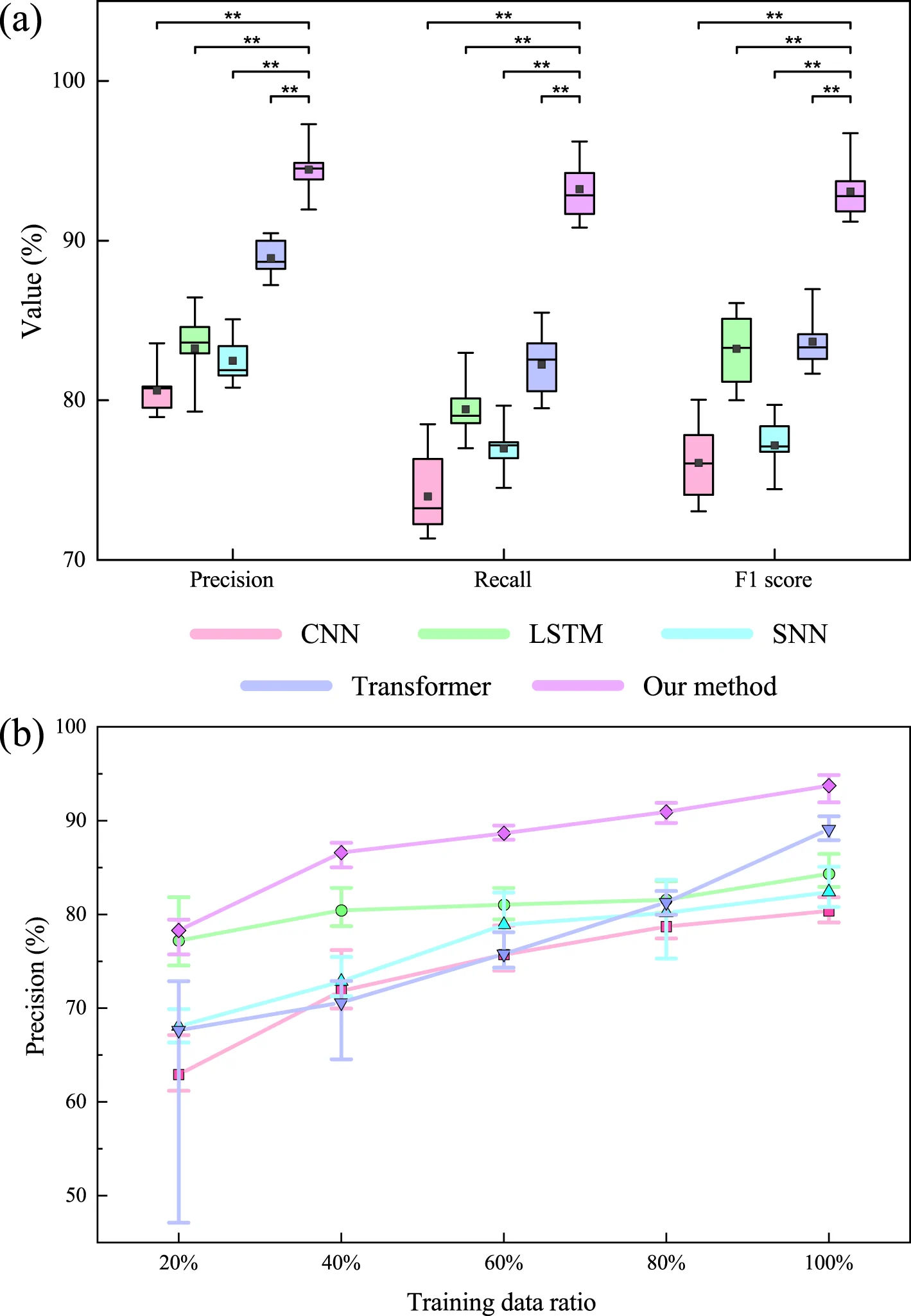
Performance comparison of the proposed SCN-MA and baseline models (CNN, LSTM, SNN, and Transformer) from statistical and data efficiency perspectives. **a** Box plots of precision, recall, and F1 score across all subjects for the proposed SCN-MA and the baseline models. Statistical significance is indicated by ** (*p < 0.01*, Bonferroni corrected). **b** Comparison of precision for different models under varying training data ratios

In addition, a statistical analysis was conducted based on the subject-wise results in Table 2 to ensure the reliability of our results. Each subject was treated as a separate dataset. Since the compared results were paired and the distribution of the differences between the two groups was not normal [43], the Wilcoxon signed-rank test was adopted to compare the proposed SCN-MA with each baseline model. Bonferroni correction was further applied to account for multiple comparisons. The box plots of precision, recall, and F1 score are presented in Fig. 12a. Statistical significance is indicated by asterisks(*), where * denotes *p< 0.0125* after Bonferroni correction and ** denotes *p < 0.01*. The results show that the proposed SCN-MA significantly outperforms all baseline models across precision, recall, and F1 score. These consistent improvements highlight the synergistic effect of the model architecture and its neuro-inspired design.

### Ablation study

The contribution of different components in SCN-MA was examined through an ablation study, in which individual modules were removed or modified and the resulting classification performance was compared. Since the proposed framework relies on multimodal input, the ablation analysis was designed to evaluate both the role of individual architectural modules and the necessity of multimodal fusion. As summarized in Table 3, the evaluated settings include the variants without CNN, without memory autapse, without ensemble policies, without pMMG, and without stretch sensor, together with the full SCN-MA model. By comparing these settings, the specific role of each component in the overall gait environment recognition performance can be clearly evaluated.

Table 3 shows that the full SCN-MA achieves the highest precision of 93.72%, while all ablated variants lead to performance degradation. Specifically, the precision decreases to 90.13%, 88.26%, and 85.67% when the CNN decoder, memory autapse, and ensemble policies are excluded, respectively. When single-modality input is used, the precision decreases to 89.55% with the stretch sensor signal only and 80.99% with pMMG signal only. These results demonstrate that both the architectural modules and the multimodal input design positively contribute to the overall gait environment recognition performance.

**Table 3:** Ablation results of SCN-MA under different model and input configurations

Case	Configuration	Precision (%)
Case I	SCN-MA without CNN	90.13
Case II	SCN-MA without memory autapse	88.26
Case III	SCN-MA without ensemble policies	85.67
Case IV	SCN-MA without pMMG	89.55
Case V	SCN-MA without stretch sensors	80.99
Case VI	Full SCN-MA	93.72

### Hardware deployment performance

To validate the practical deployability of the proposed SCN-MA framework, the model was implemented on a Zynq-based FPGA platform. The hardware setup utilized an AX7Z100B board, which integrates both processing and programmable logic units within the Zynq system-on-chip architecture.

**Fig. 13:**
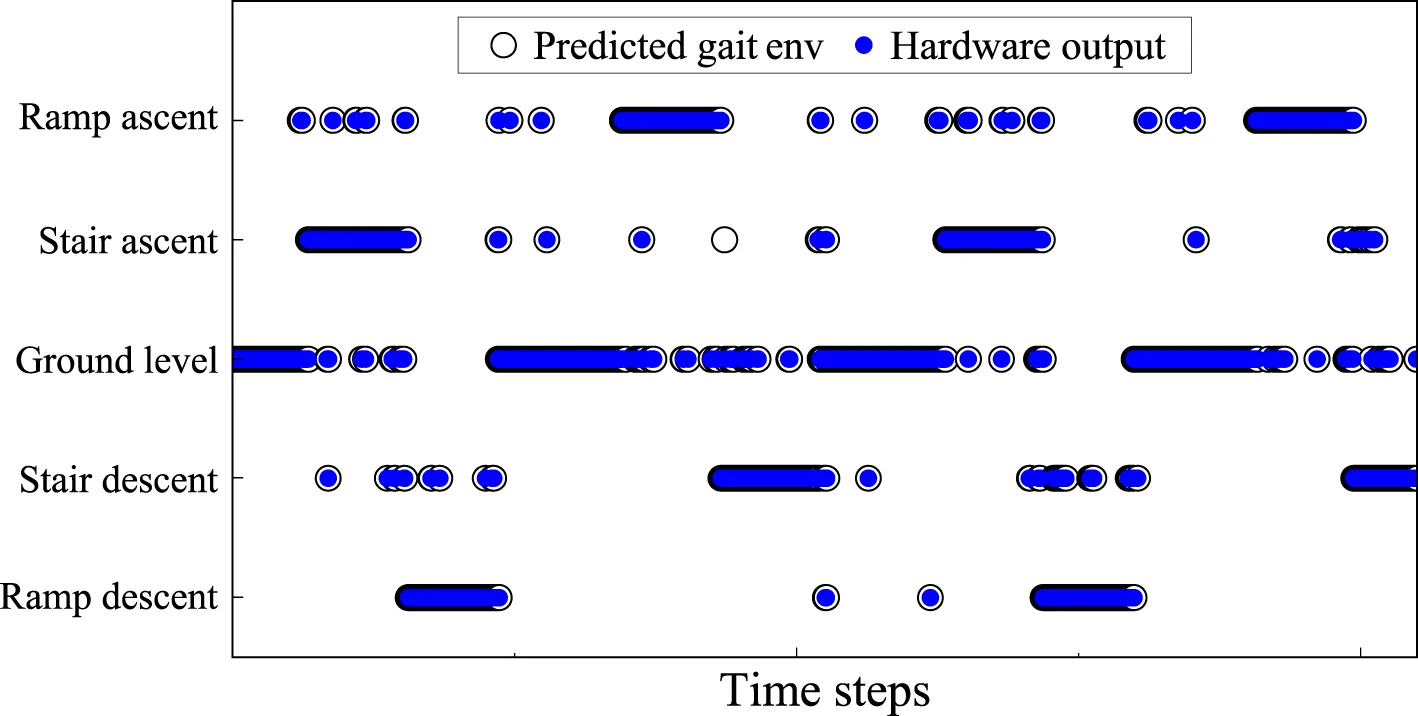
Comparison between predicted gait labels from the original model and hardware output. Strong alignment indicates that the deployed system faithfully replicates model predictions

As shown in Fig. 13, the predicted output of the hardware closely matches the predictions of the model software in all gait categories. Each row denotes one of the five gait conditions, and the alignment between hardware output and offline model predictions indicates that the proposed design maintains functional consistency after deployment. This confirms that the end-to-end neuromorphic computation pipeline has been faithfully mapped to the chip-level implementation. The system operates at 150 MHz, with an average inference latency of 0.982 ms per sample, which significantly exceeds the real-time processing requirements for wearable control systems. Power analysis shows an average dynamic power consumption of 1.87 W during operation. Table 4 summarizes the key deployment metrics.

**Table 4:** Hardware deployment metrics on FPGA board

Metric	Value
Operating frequency	150 MHz
Inference latency	0.982 ms
Power consumption	1.87 W
Logic resource usage (LUT/FF)	26.2%/17.1%
DSP usage	18.70%
BRAM usage	45.40%

## Discussion

This study proposes a neuromorphic framework for gait environment recognition that integrates multimodal sensing with a synaptic convolutional neural network featuring memory autapses. The experimental results across ten subjects demonstrate that SCN-MA consistently outperforms conventional CNN, LSTM, SNN, and Transformer baseline models. This section discusses the observed performance characteristics, architectural implications, practical relevance, and remaining limitations.

To interpret the comparative results, it is important to examine why SCN-MA outperforms the baseline models. The weaker performance of other models can be attributed to their inherent architectural limitations. Conventional CNNs, though effective in image recognition, are not well-suited for temporally dynamic signals, often overlooking subtle phase transitions. LSTM networks are capable of sequence modeling but suffer from gradient degradation over long sequences, leading to instability in recall, especially under signal variability. Although standard SNNs capture event-based dynamics efficiently, their lack of internal memory mechanisms limits their ability to detect transient features during terrain transitions. SCN-MA addresses this gap by introducing autapse dynamics into the spiking framework. The autaptic feedback enables each neuron to maintain a short-term memory trace, allowing the model to retain prior spike history and enhance discrimination across ambiguous time intervals. This mechanism, in combination with temporal excitation from synaptic currents, improves classification robustness, especially at environment boundaries. While Transformer-based models demonstrate relatively high precision, their reliance on attention layers incurs substantial computational cost. In practice, the average inference latency of Transformer models is approximately three times higher than that of SCN-MA, making them less suitable for real-time applications such as wearable robotics. In contrast, SCN-MA combines accuracy and computational efficiency through sparse spike encoding and parallel convolutional decoding, ensuring feasibility for embedded deployment. These results confirm that SCN-MA not only outperforms baseline methods in accuracy but also offers greater practicality for time-sensitive and hardware-constrained gait recognition systems.

**Fig. 14:**
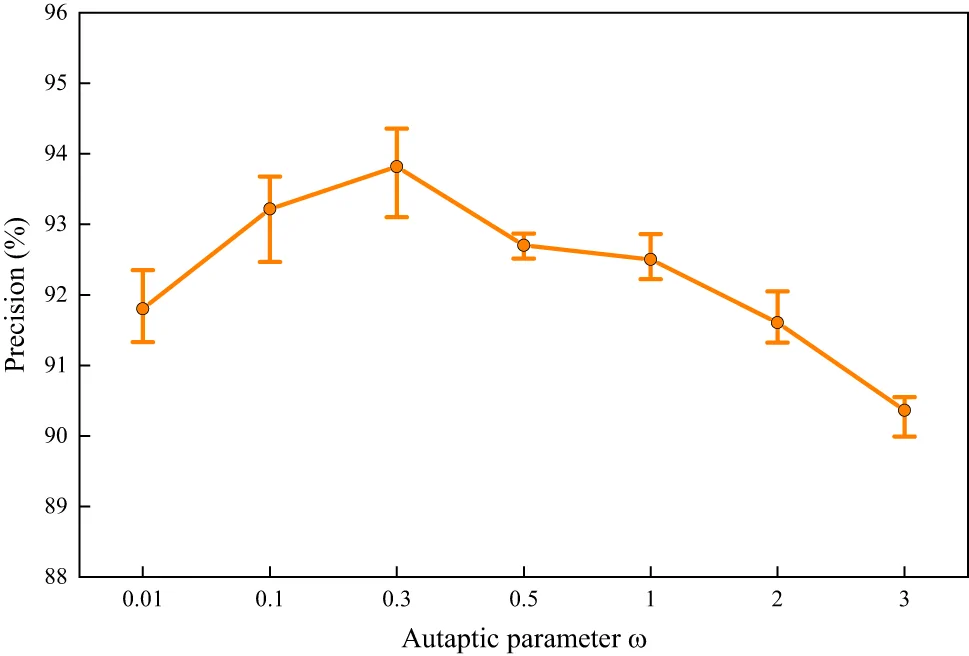
Classification precision under different values of the autaptic parameter *ω *

To further evaluate the influence of the autaptic mechanism, a sensitivity analysis was conducted on the autaptic parameter *ω *. In the proposed model, *ω * regulates the strength of recurrent self-feedback from previous output spikes to the current neuronal state, thereby directly affecting the balance between historical spiking memory and the response to current input dynamics. Although *ω * is learnable during training, it was treated as an explicit parameter in this analysis and manually assigned different values. The corresponding results are presented in Fig. 14.

As shown in Fig. 14, the classification precision first increases and then decreases as the autaptic parameter *ω * becomes larger, with the best performance obtained at *ω = 0.3* in the present experiment. When *ω * is too small, the recurrent self-feedback is insufficient, and the contribution of previous spiking activity to the current neuronal state cannot be effectively preserved. As a result, the short-term temporal dependency modeled by the memory autapse is not fully exploited. When *ω * is increased to a moderate range, useful temporal context can be retained more effectively, leading to improved gait pattern discrimination. However, when *ω * becomes excessively large, a continuous decline in precision is observed. This behavior can be attributed to excessively strong recurrent feedback, through which previous spike activity is repeatedly amplified in subsequent synaptic updates. Under this condition, neuronal responses become dominated by historical activity, while their sensitivity to current input dynamics is weakened. Consequently, the discrimination of subtle temporal differences between gait patterns is reduced. These results indicate that an excessively small *ω * leads to insufficient memory enhancement, whereas an excessively large *ω * introduces overly strong historical dependence. In this experiment, the *ω = 0.3* provides the most favorable balance between temporal memory retention and current-pattern discrimination.

The ablation analysis provides additional insight into the contribution of different components in SCN-MA. The performance decreases observed across all ablated settings suggest that the performance of SCN-MA depends on the combined effect of multiple architectural modules and multimodal input fusion. The reduced performance without the CNN decoder suggests that the convolutional module contributes to extracting locally discriminative patterns from spike-based representations and capturing short-term dynamic changes in gait signals. The decrease observed without the memory autapse suggests that this component contributes to modeling temporal dependencies in sequential gait signals. The influence of the ensemble policies is more pronounced among the architectural ablations. In Case III, excluding the ensemble policies reduces the precision to 85.67%, indicating that the policy-based fusion strategy is essential for robust final decision-making. Its contribution lies not only in combining multiple binary classifiers, but also in alleviating the influence of data imbalance. In the current data distribution, ground-level road accounts for a relatively large proportion, which may cause the corresponding gait classifier to dominate the prediction. The ensemble strategy mitigates this effect by adaptively adjusting classifier weights, thereby reducing the influence of the ground-level-related classifier when its output becomes overly dominant and improving the balance of class discrimination. The single-modality results suggest that stretch sensor signals provide stronger discriminative information than pMMG alone for gait environment recognition. Since the stretch sensors are attached around the hip, knee, and ankle joints, they can directly capture changes in joint kinematics information associated with different walking environments. By contrast, pMMG recorded from the thigh and shank muscles mainly reflects local muscle contraction dynamics, which are less directly associated with terrain categories than joint kinematic patterns. Nevertheless, the improved performance of the full SCN-MA suggests that pMMG provides complementary muscle-related information, which further improves the overall recognition performance.

The relationship between model performance and training set size was further examined to assess sample efficiency under scarce training data conditions. Figure 12b compares the precision of different models under varying training data ratios. When the training proportion is reduced to 80%, 60%, 40%, and 20% of the original training set, the performance of all models declines to different degrees. The degradation of CNN, SNN, and particularly Transformer is more evident, suggesting that these models depend more on larger training sets to learn stable gait representations. SCN-MA maintains the highest precision when the training proportion remains at or above 40%, indicating that the proposed model can retain its discriminative capability under limited-data conditions. The synaptic dynamics and memory autapse enhance temporal representation and short-term dependency modeling, which helps distinguish gait environments with overlapping transition patterns. At the same time, the decomposition of the multi-class task into multiple binary classification subtasks, together with the reinforcement learning-based ensemble strategy, helps reduce learning difficulty and improve decision stability under limited supervision. Under the most limited 20% training condition, the precision of SCN-MA decreases more noticeably and is surpassed by LSTM. This suggests that, although SCN-MA performs well in moderate small-sample settings, its performance still depends on a minimum amount of training data. Overall, these findings indicate that SCN-MA provides a favorable balance between recognition performance and sample efficiency when labeled gait data are limited but not extremely scarce.

Hardware deployment results further validate the feasibility of real-time neuromorphic inference. The resource utilization results show that the system consumes only 26.2% of the available Look-Up Tables (LUTs), 17.1% of Flip-Flops (FFs), and 18.7% of DSP slices. The relatively high usage of BRAM is attributed to buffering and spike-data storage; however, it remains within acceptable limits. The utilization results demonstrate the architecture’s scalability, allowing future expansion to additional gait classes or integrated sensory fusion without exceeding resource constraints. The results further show that SCN-MA achieves low latency, which stem from its event-driven architecture, modular binary-classification scheme, and sparse synaptic updates. The use of synaptic neurons with autapse dynamics enables temporal memory encoding without relying on large recurrent structures or attention mechanisms, resulting in more efficient computation. In summary, the hardware implementation of SCN-MA demonstrates that the model achieves high-speed inference, low latency, and competitive energy efficiency while maintaining prediction accuracy. These characteristics confirm the feasibility of deploying neuromorphic gait-environment recognition for real-time adaptive control in wearable robotic systems.

The multimodal sensing framework and SCN-MA model offer several practical benefits for rehabilitation and assistive mobility. By identifying gait environments in real time, the system enables proactive adjustments in assistive devices. For example, a powered exoskeleton could adjust hip and knee support levels when approaching stairs or ramps, allowing smoother transitions and reducing the physical demands on the user. Similarly, smart walkers or ankle–foot orthoses could adapt their stability assistance according to terrain, improving balance and reducing user effort during locomotion. These applications illustrate that the proposed framework aligns with practical needs in gait rehabilitation and community mobility. Rather than serving solely as a technical contribution, the system provides a foundation for adaptive assistance strategies that support safe and efficient movement across diverse real-world environments.

Despite promising performance, several limitations remain. The dataset includes a limited number of healthy participants, and the model has not yet been evaluated on clinical populations. Although the approach generalizes well across individuals, broader validation involving stroke survivors, spinal cord injury patients, and lower-limb amputees is needed to fully assess robustness under pathological gait variations. In addition, the experiments were conducted in controlled conditions with predefined terrain boundaries. In real-world environments, however, terrains are often more irregular, including uneven surfaces, damaged pavements, or non-uniform steps, which may introduce additional variability. The generalization capability of the proposed method to such unseen or mixed terrains has not been explicitly evaluated in this study. Based on our current results and analysis, the use of multimodal sensing and the temporal modeling mechanism in SCN-MA may contribute to improved robustness under these conditions. Since the model captures both joint kinematic patterns and muscle-related dynamics, it relies less on terrain-specific features and more on underlying gait characteristics, which may help it adapt to variations in walking conditions. Future work will focus on expanding the participant pool to include clinical populations, evaluating the model under real-world perturbations and mixed-environment scenarios. In addition, integrating neuromorphic predictions into closed-loop exoskeleton control and extending the hardware design toward fully wearable implementations will be explored to support deployment in practical rehabilitation and daily mobility scenarios.

## Conclusions

This work presents a neuromorphic framework for real-time gait environment recognition, addressing the challenges of dynamic terrain adaptation in wearable robotics. A multimodal dataset was constructed using pMMG and capacitive stretch sensors under diverse walking conditions, enabling the assessment of muscle activity and joint kinematics. Based on this dataset, a synaptic convolutional neural network with memory autapse (SCN-MA) was developed to enhance temporal representation and classification robustness. A reinforcement learning-based ensemble strategy further improves prediction accuracy across gait categories. The entire system was deployed on a Zynq-based FPGA platform, achieving end-to-end spike-based inference with low latency and high computational efficiency. Future work will further investigate cross-participant gait environment recognition to improve the subject-independent generalization capability of the proposed framework, addressing inter-subject variability in multimodal physiological signals. In addition, this framework will be extended toward closed-loop exoskeleton control by integrating prediction results into gait-adaptation mechanisms. This advancement will support the development of intelligent exoskeleton systems capable of proactive terrain response and enhanced user safety in real-world scenarios.

## Data Availability

The datasets used and/or analysed during the current study are available from the corresponding author on reasonable request.
